# Efficacy and safety of de-escalation therapy to ertapenem for treatment of infections caused by extended-spectrum-β-lactamase-producing *Enterobacteriaceae*: an open-label randomized controlled trial

**DOI:** 10.1186/s12879-017-2284-1

**Published:** 2017-03-01

**Authors:** Pinyo Rattanaumpawan, Peerawong Werarak, Anupop Jitmuang, Pattarachai Kiratisin, Visanu Thamlikitkul

**Affiliations:** 1 Division of Infectious Diseases and Tropical Medicine, Department of Medicine, Faculty of Medicine Siriraj Hospital, Mahidol University, Bangkok, Thailand; 2 Department of Preventive Medicine, Faculty of Medicine Siriraj Hospital, Mahidol University, Bangkok, Thailand; 3 Department of Microbiology, Faculty of Medicine Siriraj Hospital, Mahidol University, Bangkok, Thailand

**Keywords:** ESBL, *Enterobacteriaceae*, Carbapenem, De-escalation, Ertapenem

## Abstract

**Background:**

Carbapenem antibiotics are considered the treatment of choice for serious extended-spectrum beta-lactamase (ESBL)-producing Gram-negative bacteria (GNB) infections. The study objectives were to evaluate efficacy and safety of de-escalation therapy to ertapenem for treatment of infections caused by extended-spectrum-β-lactamase-producing Enterobacteriaceae.

**Methods:**

We conducted a randomized controlled trial of adult patients with documented ESBL-producing *Enterobacteriaceae* infections who had received any group 2 carbapenem for less than 96 h. In the intervention group, the previously-prescribed group 2 carbapenem was de-escalated to ertapenem. In the control group, the group 2 carbapenem was continued.

**Results:**

During June 2011–December 2014, 32 patients were randomized to the de-escalation group and 34 to the control group. Most common sites of infection were urinary tract infection (42%). Characteristics of both groups were comparable. By using a 15% predefined margin, ertapenem was non-inferior to control group regarding the clinical cure rate (%Δ = 14.0 [95% confidence interval: −2.4 to 31.1]), the microbiological eradication rate (%Δ = 4.1 [−5.0 to 13.4]), and the superimposed infection rate (%Δ = −16.5 [−38.4 to 5.3]). Patients in the de-escalation group had a significantly lower 28-day mortality rate (9.4% vs. 29.4%; *P* = .05), a significantly shorter median length of stay (16.5 days [4.0–73.25] vs. 20.0 days [1.0–112.25]; *P* = .04), and a significantly lower defined daily dose of carbapenem use (12.9 ± 8.9 vs. 18.4 ± 12.6; *P* = .05).

**Conclusions:**

Ertapenem could be safely used as de-escalation therapy for ESBL-producing *Enterobacteriaceae* infections, once the susceptibility profiles are known. Future studies are needed to investigate ertapenem efficacy against ESBL-producing *Enterobacteriaceae* pneumonia to determine its applicability in life-threatening conditions.

**Trial registration:**

ClinicalTrials.gov identifier: NCT01297842. Registered on 14 February 2011. First patient enrolled on 27 June 2011.

## Background

Empirical broad-spectrum antimicrobial therapy is highly recommended in patients with serious infections in aiming to improve patient’s outcomes [1]. However, overuse of broad spectrum antimicrobial therapy is an important risk factor for the emergence of bacterial resistance [2]. De-escalation therapy has been proposed as a strategy for balancing the advantages of adequate empirical therapy with the risk of emergence of bacteria resistance [3]. De-escalation therapy involves the initial use of empirical broad-spectrum antimicrobial therapy, which is then streamlined to more narrow-spectrum or targeted agents once culture and susceptibilities are available [4]. Efficacy of de-escalation therapy in patients with severe infections has been confirmed in many previous studies [5–7].

There are two carbapenem groups currently available worldwide: group 1 carbapenem (i.e.,ertapenem) and group 2 carbapenems (i.e., imipenem, meropenem, doripenem, and biapenem). Group 2 carbapenems are potent antibiotics that act against Gram-positive bacteria and Gram-negative bacteria, including extended-spectrum beta-lactamase-producing *Enterobacteriaceae*, *Pseudomonas aeruginosa*, and *Acinetobacter* spp*.* As compared to group 2 carbapenems, ertapenem is not active against *P. aeruginosa* and *Acinetobacter* spp. [8]. Nevertheless, a recent systematic review did not find a significant difference in resistant *Enterobacteriaceae* colonization between patients who received ertapenem and patients who received group 2 carbapenems [9].

Carbapenem antibiotics are considered the treatment of choice for serious infections caused by ESBL-producing *Enterobacteriaceae* [10]. Many studies have reported that ertapenem has promising efficacy as definitive therapy for these serious infections, but the majority of these studies were observational studies [11–13]. Based on results from a recent propensity score-matching cohort study, ertapenem appears as effective as group 2 carbapenems for empirical and definitive therapy of bloodstream infections caused by ESBL-producing *Enterobacteriaceae* [14].

Group 2 carbapenems have to be administered parenterally at least twice a day, while ertapenem is generally administered once daily and can be given by intramuscular injection [8]. However, the maximum serum concentration of ertapenem is slightly lower after intramuscular administration compared with intravenous administration [15].

Therefore, some clinically stable patients could receive ertapenem as outpatient parenteral antibiotic therapy, which would have the effect of reducing both length of stay and hospital expenditure.

Given these considerations, an open-label randomized controlled trial was conducted in patients with documented ESBL-producing *Enterobacteriaceae* infections who had received group 2 carbapenems as empirical therapy. The aim was to compare the efficacy and safety of de-escalation therapy to ertapenem versus continuation of group 2 carbapenem antibiotics. Efficacy, resource use, and impact on emergence of bacterial resistance were investigated and determined.

## Methods

### Setting and study design

A single-center, open-label, randomized equivalence trial was conducted from June 2011 to December 2014 at Siriraj Hospital, a 2200-bed tertiary care university hospital in Bangkok, Thailand.

### Study subjects

Eligible subjects were hospitalized patients aged 18 years or older who had documented ESBL-producing *Enterobacteriaceae* infections and who received group 2 carbapenems as empirical therapy. Exclusion criteria were, as follows: treatment with a group 2 carbapenem for longer than 96 h; having active *P. aeruginosa* co-infection; pregnancy; breast-feeding; having a history of carbapenem hypersensitivity; and having infection caused by carbapenem-resistant *Enterobacteriaceae* strain.

### Randomization and treatment

Eligible subjects were 1:1 allocated to the intervention group or the control group by stratified randomization according to the site of infection (lower respiratory tract infection, urinary tract infection (UTI), and bacteremia). Centers for Disease Control and Prevention/National Healthcare Safety Network (CDC/NHSN) surveillance definitions for specific types of healthcare-associated infections (HAIs) were used for diagnosis of each type of infection [16]. Randomization numbers were computer-generated with permuted blocks (block size of four).

For patients in the intervention group (de-escalation group), the previously prescribed group 2 carbapenem was de-escalated to ertapenem. For patients in the control group, the group 2 carbapenem was continued for the entire course of therapy. Carbapenem dosage was adjusted according to patient renal function, as shown in the products’ package insert. Renal function was calculated using Cockcroft-Gault formula. Each dose of either gr.1 or gr.2 carbapenem was given by infusion for 1 h. Extended infusion or continuous infusion of carbapenem was not allowed in this study. Duration of systemic antibiotic therapy was determined by each patient’s attending physicians. All other non-carbapenem antibiotics were given as needed.

### Microbiological testing

Microbiological tests were routinely processed at our hospital’s microbiology laboratory. All tests were processed by a Vitek® 2 automated ID/AST instrument (bioMerieux SA, Marcy-l'Étoile, France). ESBL production was confirmed by double-disk method, according to the performance standards for antimicrobial susceptibility testing established by the Clinical and Laboratory Standards Institute (CLSI) [17].

Clinical specimens from the documented site of infection were obtained on day 3, day 7, day 14, and then at least once a week thereafter for microbiological culture. Specimen collection was discontinued if the culture result was negative or the course of antibiotic treatment was completed. To determine colonization by resistant Gram-negative pathogens, a stool culture or a rectal swab culture was obtained on day 0, at the end of therapy, and on day 28 of therapy if there were no contraindications. The target resistant Gram-negative pathogens were ESBL-producing *Enterobacteriaceae*, multidrug-resistant (MDR) *P. aeruginosa*, and MDR *A. baumannii*.

### Data collection and outcome assessment

Baseline data, including demographic characteristics, underlying diseases, Acute Physiology and Chronic Health Evaluation II (APACHE II) score, intensive care unit (ICU) admission, and previous antibiotic therapy were collected. Clinical outcomes were evaluated on a daily basis. Cost-related data, including length of stay (LOS) and amount of drug therapy for ESBL-producing *Enterobacteriaceae* were also collected. All adverse events were recorded for 28 days from the date of enrollment.

The primary outcome was clinical cure rate at the end of therapy. Secondary outcomes included microbiological eradication rate, superimposed infection rate during study treatment, 28-day mortality rate, and adverse drug reactions (ADRs). Prevalence of colonization with resistant pathogens and economic outcomes (LOS, antibiotic consumption, and hospital expenditure) were also evaluated. Study definitions are shown in Table 1.

**Table 1:** Study definitions

Terms	Definitions
Clinical cure	Resolution of or improvement in the index infection without need for further antibiotic therapy
Microbiological eradication	At least one negative culture of an index pathogen from a clinical specimen that was obtained during or after the course of antibiotic therapy
Superimposed infection	New onset of infection caused by any pathogens other than ESBL-producing *Enterobacteriaceae* during the course of antibiotic therapy
28-day mortality	Death from any cause within 28 days after enrollment

### Statistical analysis

Based on data obtained from our pilot study [unpublished data], the estimated clinical cure rate of group 2 carbapenems in the treatment of ESBL-producing *Enterobacteriaceae* infection is approximately 90%. To test the equivalency hypothesis with a predefined margin of ±15% using a 2-sided confidence interval (CI) approach, the required sample size was calculated to be 100 patients or 50 patients for each of two groups. However, the study was terminated early due to slow recruitment of eligible subjects. As such, this study describes an interim analysis of 66 enrolled patients. Data are presented as *n* (%), mean ± standard deviation (SD), or median [range]. Treatment outcomes are reported as % difference (%Δ, % of the de-escalation group - % of the control group) and 95% CI. Intention to treat analysis was used for all analyses. All statistical calculations were performed using STATA version 14.0/IC (StataCorp LP, College Station, TX, USA).

## Results

**Fig. 1:**
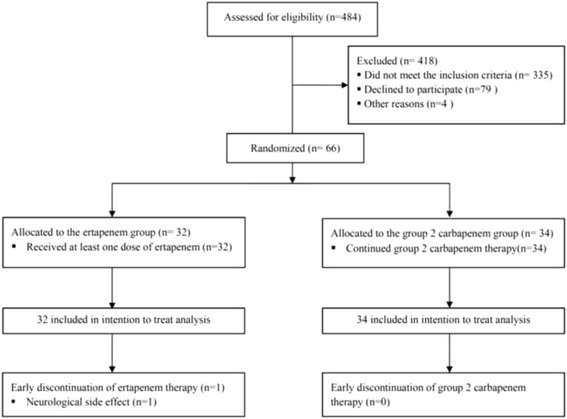
Study flow chart

From June 2011 to December 2014, 484 patients with documented ESBL-producing *Enterobacteriaceae* infection were empirically treated with group 2 carbapenems at our medical center. Sixty-six of those patients satisfied the inclusion criteria and were enrolled in this study (Fig. 1). Thirty-two patients were randomized into the de-escalation group (intervention group) and 34 into the non-de-escalation group (control group). De-escalation group patients were de-escalated to ertapenem and non-de-escalation group patients were continued with their previously prescribed group 2 carbapenem. Demographic and clinical characteristics between groups were comparable (Table 2). Nearly 60% of patients were female, with a mean age of 64.8 years (67.5 ± 17.2 years in the de-escalation group and 62.2 ± 21.6 years in the non-de-escalation group).

**Table 2:** Characteristics of patients in the de-escalation group (intervention) and in the non-de-escalation group (control)

Variables	All (*n* = 66)	De-escalation (*n* = 32)	Non-de-escalation (*n* = 34)	*P*-value
Baseline characteristics				
Mean age, years	64.8 ± 19.6	67.5 ± 17.2	62.2 ± 21.6	0.28
Female	38 (57.6%)	19 (59.4%)	19 (55.9%)	0.81
Medicine ward	58 (87.9%)	26 (81.3%)	32 (94.1%)	0.11
Underlying disease				
● Hypertension	37 (56.1%)	19 (59.4%)	18 (52.9%)	0.60
● Cerebrovascular disease	15 (22.7%)	9 (28.1%)	6 (17.7%)	0.31
● Chronic lung disease	10 (15.2%)	2 (6.3%)	8 (23.5%)	0.08
● Cardiovascular disease	22 (33.3%)	11 (34.4%)	11 (32.4%)	0.86
● Diabetes mellitus	25 (37.9%)	11 (34.4%)	14 (41.2%)	0.57
● Chronic kidney disease	23 (34.9%)	12 (37.5%)	11 (32.4%)	0.66
● Chronic liver disease	7 (10.6%)	3 (9.4%)	4 (11.8%)	1.00
● Malignancy	17 (26.6%)	9 (28.1%)	8 (23.5%)	0.67
● Any immunocompromised condition^a^	16 (24.2%)	7 (21.9%)	9 (36.5%)	0.66
APACHE II score at enrollment^b^	12.4 ± 5.9 (*n* = 10)	13.8 ± 8.2 (*n* = 5)	11.0 ± 2.3 (*n* = 5)	0.49
Modified APACHE II score at enrollment^c^	8.9 ± 4.9	8.9 ± 5.1	8.9 ± 4.9	0.94
● History of organ insufficiency	35 (53.9%)	20 (62.5%)	15 (45.5%)	0.17
● Emergency surgery	8 (12.1%)	5 (15.6%)	3 (8.8%)	0.47
● Acute renal failure	8 (12.1%)	8 (25.0%)	0 (0.0%)	0.002
● ICU admission	3 (4.6%)	2 (6.3%)	1 (2.9%)	0.42
Characteristics of infections				
Hospital-acquired infection	32 (48.5%)	12 (37.5%)	20 (58.8%)	0.08
Site of infection at enrollment				1.00
● Urinary tract infection	27 (40.9%)	13 (40.6%)	14 (41.2%)	
● Pneumonia	11 (16.7%)	5 (15.6%)	6 (16.7%)	
● Others	29 (43.9%)	14 (43.8%)	15 (44.1%)	
● Primary bacteremia	26 (78.8%)	14 (82.4%)	12 (75.0%)	
● Skin infection	1 (1.5%)	0	1 (2.9%)	
● Reproductive tract infection	1 (1.5%)	0	1 (2.9%)	
● Gastrointestinal tract infection	1 (1.5%)	0	1 (2.9%)	
Having bacteremia	33 (50.0%)	17 (53.1%)	16 (47.1%)	0.62
Cause of bacteremia^d^		(*n* = 17)	(*n* = 16)	0.57
● Primary bacteremia	26 (78.8%)	14 (82.4%)	12 (75.0%)	
● Urinary tract infection	5 (15.2%)	3 (17.6%)	2 (12.5%)	
● Catheter-related infection	2 (6.0%)	0	2 (12.5%)	
Causative pathogen^e^				
● *Escherichia coli*	46 (69.7%)	24 (75.0%)	22 (64.7%)	0.36
● *Klebsiella* spp.	21 (31.8%)	8 (25.0%)	13 (38.2%)	0.25
Mean time from onset of infection to enrollment, days	4.3 ± 1.4	4.2 ± 1.6	4.5 ± 1.2	0.47
Mean duration of pre-enrollment use of group 2 carbapenems, days	2.8 ± 1.6	3.1 ± 1.5	2.6 ± 1.7	0.29
Mean duration of pre-enrollment use of any antibiotics, days	3.1 ± 5.8	3.3 ± 5.4	3.0 ± 6.3	0.82

^a^Any immunocompromised condition (e.g., HIV infection, receipt of immunosuppressive agents (steroid, cytotoxic agents, and/or chemotherapy))

^b^APACHE II score was calculated using data from 10 patients with available arterial blood gas results

^c^Modified APACHE II score was calculated in all patients. For those who did not have arterial blood gas results, the arterial pH was substituted by the level of serum bicarbonate

^d^All patients with secondary bacteremia due to UTI (*n* = 5) were enrolled into the UTI stratum. Other cases of secondary bacteremia were due to cellulitis (*n* = 1) and spontaneous bacterial peritonitis (*n* = 1)

^e^One patient in the group 2 carbapenems group had bacteremia caused by both *Escherichia coli* and *Klebsiella* spp

*Abbreviation*: *APACHE II* Acute Physiology and Chronic Health Evaluation II

Data are presented as n (%), mean ± SD; *P*-value < 0.05 indicates statistical significance

Twelve patients (37.5%) in the de-escalation group and 20 patients (58.8%) in the non-de-escalation group had a healthcare-associated infection (*P =* .08). Approximately 40% of patients in both groups had urinary tract as the site of ESBL-producing *Enterobacteriaceae* infection. Fourteen patients in the de-escalation group (43.8%) and 12 patients in the non-de-escalation group (35.3%) had primary bacteremia. The mean APACHE II score and mean modified APACHE II score were comparable between groups. Mean time from onset of infection to enrollment was 4.2 ± 1.6 days in the de-escalation group and 4.5 ± 1.2 days in the non-de-escalation group *(P =* .47). There was no significant difference in duration of pre-enrollment use of group 2 carbapenems (3.1 ± 1.5 days *vs.* 2.6 ± 1.7 days; *P* = .29) or duration of pre-enrollment use of any other antibiotic (3.3 ± 5.4 days *vs.* 3.0 ± 6.3; *P* = .82) between the de-escalation and non-de-escalation groups, respectively.

Clinical, microbiological and cost-related outcomes of patients in the de-escalation group (intervention) and in the non-de-escalation group (control) are shown in Table 3. This study failed to prove equivalency in all clinical outcomes, including clinical cure rate (%Δ = 14.0 [95% CI: -2.4 to 31.1]), microbiological eradication rate (%Δ = 4.1 [-5.0 to 13.4]), superimposed infection (%Δ = -16.5 [-38.4 to 5.3]), and 28-day mortality (%Δ = -20.0 [-39.3 to -0.8]). Although the equivalency hypothesis was not proven, the lower bounds of the CIs for difference in clinical cure and microbiological eradication rate were within the -15% margin. The upper bounds of the CIs for difference in 28-day mortality and superimposed infection were within the +15% margin. Therefore, non-inferiority of de-escalation was established for all primary and secondary outcomes. Additionally, the upper bound of the CI for difference in 28-day mortality was below zero, probably suggesting the superiority of de-escalation for 28-day mortality.

**Table 3:** Outcomes of patients in the de-escalation group (intervention) and in the non-de-escalation group (control)

Outcomes	De-escalation (*n* = 32)	Non-de-escalation (*n* = 34)	*P*-value
Clinical outcomes			
Clinical cure rate	30 (93.8%)	24 (79.4%)	0.09
Microbiological eradication rate^a^	20/20 (100.0%)	23/24 (95.8%)	0.36
28-day mortality rate	3 (9.4%)	10 (29.4%)	0.05^b^
• ID-related mortality	1 (3.1%)	9 (26.5%)	0.01^b^
• Non ID-related mortality	1 (3.1%)	1 (2.9%)	1.00^b^
Superimposed infection rate	6 (18.8%)	12 (35.3%)	0.13
• *Acinetobacter baumannii*	2 (6.3%)	6 (17.3%)	0.26^b^
• MRSA	2 (6.3%)	3 (8.8%)	1.00^b^
• *Candida* spp.	1 (3.1%)	4 (11.8%)	0.51^b^
• *Pseudomonas aeruginosa*	2 (6.3%)	2 (5.9%)	1.00^b^
• *Enterococcus *spp.	2 (6.3%)	0	0.23^b^
Cost-related outcomes			
Median length of stay, days	16.5 (4.0–73.0)	20.0 (1.0–112.0)	0.04^b^
Median length of stay after enrollment, days	9.5 (0–37.0)	11.5 (1.0–102.0)	0.11^b^
Mean duration of carbapenem use, days	14.4 ± 6.0	16.5 ± 10.5	0.32
Mean DDD of carbapenem	12.9 ± 8.9	18.4 ± 12.6	0.05
Stool colonization	*N* = 32	*N* = 32^c^	
At baseline			
• Any MDR bacteria	20 (62.5%)	21 (65.6%)	1.0
• Any ESBL-producing GNB	19 (59.4%)	20 (62.5%)	1.0
• MDR-*Acinetobacter baumannii*	4 (12.5%)	3 (9.4%)	1.0^b^
• MDR-*Pseudomonas aeruginosa*	0	1 (3.1%)	1.0^b^
At the end of therapy			
• Any MDR bacteria	16 (50.0%)	11 (34.4%)	0.31
• Any ESBL-producing GNB	11 (34.4%)	8 (25.0%)	0.59
• MDR-*Acinetobacter baumannii*	6 (18.8%)	4 (12.5%)	0.73^b^
• MDR-*Pseudomonas aeruginosa*	1 (3.1%)	0 (0.0%)	1.0^b^

^a^Microbiological eradication was calculated in patients who had a follow-up culture of the infection site

^b^Nonparametric test

^c^Two patients in the group 2 carbapenem group had a contraindication for rectal swab culture

*Abbreviations*: *MRSA* methicillin-resistant *Staphylococcus aureus*, *DDD* defined daily dose, *MDR* multi-drug resistant, *ESBL* extended-spectrum beta-lactamase enzyme, *GNB* Gram-negative bacteria

Data are presented as n (%), mean ± SD or median (range); *P*-value < 0.05 indicates statistical significance

Superimposed infection rate was slightly lower among patients in the de-escalation group (18.8% vs. 35.3%; *p* = 0.13). The three leading causative pathogens of superimposed infection were MDR *A. baumannii*, methicillin-resistant *Staphylococcus aureus*, and *Candida* spp. There was no significant difference in the distribution of causative pathogens between groups. Patients in the de-escalation group had a significantly shorter median length of hospital stay (16.5 days [4.0-73.0] *vs.* 20.0 days [1.0-112.0]; *p* = .04) and a significantly lower defined daily dose (DDD) of carbapenem antibiotic use (12.9 ± 8.9 *vs.* 18.4 ± 12.6; *p* = .05). There were no significant differences in stool colonization by MDR pathogens at enrollment or at the end of therapy.

Ertapenem was discontinued early in one patient due to the development of neurological side effects, including dizziness and headache. All neurological symptoms subsided after ertapenem was switched to meropenem. There was no early discontinuation in the non-de-escalation group that related to adverse drug reaction (ADRs). There was no statistical difference in ADRs between groups.

## Discussion

The findings of this study indicate that de-escalation therapy to ertapenem is non-inferior to continuation of group 2 carbapenems for clinical cure rate, microbiological eradication rate, and superimposed infection rate. These findings confirm the efficacy of ertapenem and are consistent with the findings of previously documented in many observational cohort studies [11–14]. The slightly lower rate of superimposed infections among patients in the de-escalation group also supported the findings of previous studies that ertapenem has lower collateral impact. Further, the lower rate of superimposed infections may be a contributing factor to the significantly lower 28-day mortality rate among patients in the de-escalation group. Given that the patients in the de-escalation group were slightly older and sicker than those in the non-de-escalation group, the lower 28-day mortality among the de-escalation group was unlikely to be the results of unbalanced baseline characteristics.

Similar to results from a recent systematic review, our study did not find any significant difference in stool colonization by MDR pathogens. However, our baseline rate of colonization with resistant GNB pathogen was relatively high (>60%), compared with the studies included in another systematic review [9].

Patients in the de-escalation group also had a significantly shorter length of hospital stay, as well as a significantly lower defined daily dose (DDD) of carbapenem use. This can be simply explained by the practice of once-daily dosing of ertapenem [8], whereby some clinically stable patients could be discharged early and treated with parenteral antimicrobial therapy as outpatients.

This study has several inherent strengths. While most previous studies were observational, this was a randomized controlled trial. Randomization ensured a balance of measured and unmeasured confounders between the intervention and control groups. In addition to the evaluation of clinical outcomes, other important parameters, including stool colonization with resistant GNB and cost-related outcomes were evaluated.

This study also has some mentionable limitations. First, participation bias and observer bias may have occurred because the study design was open-label. However, the microbiological cure rate and 28-day mortality rate are objective measures, so observer bias would have been minimal. Second, the majority of patients in this study had UTI or primary bacteremia, with only a small proportion having pneumonia. Thus, we are not able to conclude that de-escalation to ertapenem is effective against ESBL-producing *Enterobacteriaceae* pneumonia. Third, the study was early terminated before achieving the target sample size, therefore all statistically significant findings are less reliable. Lastly, the study was designed to stratify only on the site of infection but did not stratify on other clinically significant factors such as age and APACHE score. Nevertheless, these two important factors were comparable between two groups.

## Conclusions

Based on the results of this study, ertapenem can be safely used as de-escalation therapy for ESBL-producing *Enterobacteriaceae* infections, especially UTIs and primary bacteremia. Future studies are needed to investigate ertapenem efficacy against ESBL-producing *Enterobacteriaceae* pneumonia to determine its applicability in life-threatening conditions. Additionally, a study in the cost-effectiveness of de-escalation to ertapenem would be useful for healthcare policymakers.

## Abbreviations

APACHE II: Acute physiology and chronic health evaluation II
CDC/NHSN: Centers for disease control and prevention/National healthcare safety network
CI: confidence interval
CLSI: Clinical and laboratory standards institute
DDD: defined daily dose
ESBL: extended-spectrum beta-lactamase
GNB: Gram-negative bacteria
HAIs: Healthcare-associated infections
ICU: intensive care unit
LOS: length of stay
MDR: multidrug-resistant
UTI: urinary tract infection
